# Differences in the gray-to-white matter ratio according to different computed tomography scanners for outcome prediction in post-cardiac arrest patients receiving target temperature management

**DOI:** 10.1371/journal.pone.0258480

**Published:** 2021-10-14

**Authors:** Jae Hun Oh, Seung Pill Choi, Jong Ho Zhu, Soo Hyun Kim, Kyu Nam Park, Chun Song Youn, Sang Hoon Oh, Han Joon Kim, Sang Hyun Park

**Affiliations:** 1 Department of Emergency medicine, College of Medicine, The Catholic University of Korea, Eunpyeong St. Mary’s Hospital, Seoul, Republic of Korea; 2 Department of Emergency medicine, College of Medicine, The Catholic University of Korea, Seoul St. Mary’s Hospital, Seoul, Republic of Korea; 3 Department of Emergency medicine, College of Medicine, The Catholic University of Korea, Yeouido St. Mary’s Hospital, Seoul, Republic of Korea; University of the Basque Country, SPAIN

## Abstract

The gray-to-white matter ratio (GWR) has been used to identify brain damage in comatose patients after cardiac arrest. However, Hounsfield units (HUs), the measurement of brain density on computed tomography (CT) images, may vary depending on the machine type or parameter. Therefore, differences in CT scanners may affect the GWR in post-cardiac arrest patients. We performed a retrospective study on comatose post-cardiac arrest patients who visited the hospital from 2007 to 2017. Two CT, Lightspeed and SOMATOM, scanners were used. Two observers independently measured the HUs of the caudate nucleus, putamen, posterior internal capsule, and corpus callosum using regions of interest. We compared the GWR calculated from the HUs measured at different CT scanners. The analysis of different scanners showed statistically significant differences in the measured HUs and GWR. The HUs and GWR of Lightspeed were measured lower than SOMATOM. The difference between the two CT scanners was also evident in groups divided by neurological prognosis. The area under the curve of the receiver operating characteristic curve to predict poor outcomes of Lightspeed was 0.798, and the cut-off value for 100% specificity was 1.172. The SOMATOM was 0.855, and the cut-off value was 1.269. The difference in scanners affects measurements and performance characteristics of the GWR in post-cardiac arrest patients. Therefore, when applying the results of the GWR study to clinical practice, reference values for each device should be presented, and an integrated plan should be prepared.

## Introduction

Target temperature management (TTM) is commonly applied to patients who remain comatose following resuscitation after cardiac arrest since it has been shown to improve survival and neurological outcomes [1, 2]. Various tools for evaluating and predicting neurological prognosis following cardiac arrest have been studied, from biomarker to imaging [2]. Electroencephalogram analysis, serum neuron-specific enolase, somatosensory evoked potentials, brain diffusion magnetic resonance imaging (MRI), and the brainstem response have been presented as prognostic predictors in recent studies [2]. Another prognostic factor, the gray-to-white matter ratio (GWR), began to be used as a prognostic factor for comatose post-cardiac arrest patients after Torbey et al. quantitatively measured the difference between gray matter (GM) and white matter (WM) and has since been studied in various ways by other researchers [3–10].

In the event of cardiac arrest, the patient is placed in a deep hypoxic state. Hypoxia causes lactic acidosis, and a reduced pH causes brain swelling. In computed tomography (CT) images, brain swelling is difficult to distinguish the central sulcus or cisterns, and the ventricle size seems to have decreased [11–16]. In addition, this ischemic change appears to reduce the differentiation between GM and WM. The GWR can present patient prognosis prediction criteria by representing qualitative interpretations in quantitative value.

Some studies were conducted on a single scanner with various CT scanner settings, some were conducted with various types of machines, and others provided no details on scanner type or settings [3–10]. Additionally, brain CT images are affected by several parameters, such as voltage, current, and software version [17]. The values obtained from these different factors are likely to show a clear difference [17, 18]. Therefore, it is necessary to assume that there is no intermachine variability when analyzing the results obtained from different machines and settings. However, to our knowledge, whether these inconsistencies lead to differences in the clinically has never been analyzed.

Researchers have analyzed the brain CT images of normal adults and proved that the Hounsfield units (HUs) values of images obtained from three different machines are different. It was also concluded that the GWR is also affected by inter-scanner variability [19]. The purpose of this study was to analyze differences between the HUs and GWR of two machines in patients who received TTM after recovering from cardiac arrest and understand what these differences could indicate with regards to predicting neurological outcomes.

## Materials and methods

### Subjects

After deliberation, this study was approved by the Local Ethics Committee of the Catholic University of Korea, Yeouido St. Mary’s Hospital (SC18RESI0068). The medical records of patients who received TTM were reviewed from April 2007 to July 2017. The subjects included in the analysis were adults (≥ 18 years old) patients who received TTM after cardiopulmonary resuscitation and survived until discharge. Patients were excluded if they did not have a brain CT scan performed, had a CT with a contrast agent, died during TTM, suffered structural changes in the brain due to trauma, surgery or cerebrovascular disease, were transferred from other hospitals, or had too many artifacts caused by associated implants or accessories. A total of 86 patients were identified from medical records. Patients were evaluated for age, sex, witnessed the event, initial cardiac rhythm, initial Glasgow coma scale, location of cardiac arrest (in-hospital or out-hospital), the time between CT and arrest, and neurologic outcome at discharge.

This hospital used two CT scanners (CT 1; Lightspeed VCT, GE Healthcare, UK; and CT 2; SOMATOM Definition Flash, Siemens Healthcare, Germany). This study was retrospective, so each CT scanner’s setting channel, current, and voltage were not performed identically. TTM was maintained for 24 hours by lowering the body temperature to 33.0 ± 1°C using an external cooling device with a standardized protocol. Rewarming was performed slowly for more than eight hours until reaching normal body temperature. There were no patients who received extracorporeal membrane oxygenation treatment. Patients who received all treatments were hospitalized in the intensive care unit and received standard intensive care and monitoring, including mechanical ventilation, central venous catheters, and alternative catheters. Patients received brain CT scans as soon as possible when vital signs had stabilized after ROSC. In addition, all patients used a dedicated CT scanner installed in the emergency room. The neurological outcome of the patients was evaluated by the cerebral performance category (CPC) at the time of discharge, and 1 and 2 were defined as good outcomes. The CPC was evaluated by experts at least 72 hours after the ROSC and at discharge.

### Density measurements from the image

Two investigators analyzed images of patients. Investigator 1 is an emergency medicine specialist with over twenty years of experience, and investigator 2 is an emergency medicine specialist with eight years of experience. A radiologist trained the two researchers in related research knowledge for this study. Images were analyzed using commercial image viewing software (Marotech; M-view) that had blinded patient information. The level corresponding to the basal ganglia (BG) was determined by investigator 1, and both investigators measured the images of all patients. The two researchers did not share the results of the measurements, and the measurements were taken independently of each other. The density value was measured at 0.1 cm^2^ wide, and the average HUs value of the area with a circular region of interest (ROI) was used for analysis. The HUs of the caudate nucleus (CN), putamen (PU), posterior internal capsule (PIC), and corpus callosum (CC) on both sides were measured. As in previous studies, to increase the objectivity of the inspection, color mapping can be implemented in the image viewing software, making each area easier to visualize [19]. The area was divided into imaginary lines made using several landmarks, and the average value was defined as the HUs of the area after measurement [19]. We averaged nine values by dividing three sections per area and measuring three ROIs for each section. The mean HUs value was calculated from the CN/PIC, PU/PIC, CN/CC, and PU/CC between single areas. The GWR-AVE, including all values, was defined as (CN+PU)/(PIC+CC).

### Statistical analysis

Categorical variables are expressed as numbers and percentages. Continuous variables are expressed as the mean ± standard deviation or median and interquartile range (IQR). Chi-squared and Fisher’s exact tests were used to analyze categorical variables. Continuous variables were tested for normality through the Shapiro-Wilk test. Most continuous variables did not follow a normal distribution, so the average value between the two CT groups was assessed with the nonparametric Mann-Whitney U test. The differences between each group were compared with box and whisker plots. Interclass correlation coefficients (ICCs) were analyzed to evaluate the consistency of measurements between the two investigators. A receiver operating characteristic (ROC) curve was created to obtain a cut-off value for predicting poor outcomes. The cut-off values at 100% specificity were analyzed and compared. Two-sided p-values less than 0.05 were considered statistically significant. Statistical analysis was performed with SPSS ver. 20 (IBM Corp., Armonk, NY, USA) and MedCalc ver. 15.2.2 (MedCalc Software, Mariakerke, Belgium).

## Results

**Fig 1:**
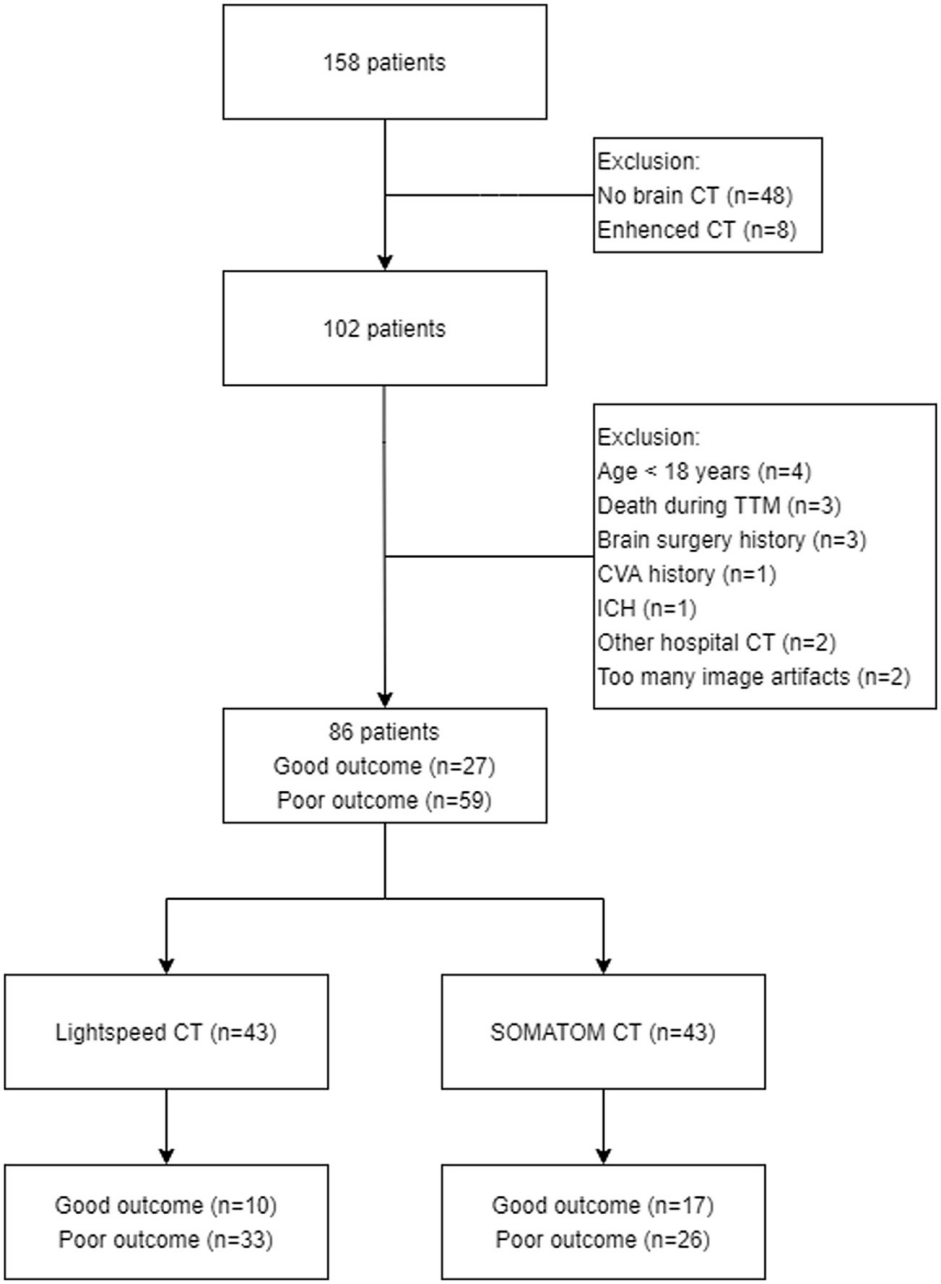
Flow diagram of the study population. CT, computer tomography; TTM, target temperature management; CVA, cerebrovascular accident; ICH, intracranial hemorrhage; CT 1, LightSpeed VCT; CT 2, SOMATOM definition flash.

Eighty-six patients were enrolled. Forty-three patients were classified into the Lightspeed group, and others were classified into the SOMATOM group (Fig 1). The median time from cardiac arrest to the CT scan was 1.2 hours (IQR, 0.8–2.6 hours), the mean patient age was 56.9 years (SD, ±16.9), and there were twenty-eight (33%) women. Fifty-five patients (64%) experienced witnessed cardiac arrest, and twenty-seven (31%) had a good neurologic outcome. All of the baseline variables showed no statistically significant differences between CT groups (Table 1).

**Table 1:** Demographic characteristics.

	All (n = 86)	Lightspeed (n = 43)	SOMATOM (n = 43)	p-value
Age, years	56.9 ± 16.9	55.5 ± 19.3	58.4 ± 14.2	0.436
Female	28 (32.6)	15 (34.9)	13 (30.2)	0.818
Witnessed	55 (64.0)	26 (60.5)	29 (67.4)	0.654
Initial rhythm				0.158
Shockable rhythm	18 (20.9)	7 (16.3)	11 (25.6)	
Non-shockable rhythm	67 (78.0)	36 (83.7)	31 (72.1)	
Unknown	1 (1.2)	0 (0)	1 (2.3)	
Initial GCS	3 [3–7]	3 [3–6]	3 [3–7]	0.169
Out of Hospital	84 (97.7)	42 (97.7)	42 (97.7)	1.000
Cause of arrest				0.369
Cardiac	39 (45.3)	16 (37.2)	23 (53.5)	
Respiratory	34 (39.5)	21 (48.8)	13 (30.2)	
Unknown	13 (15.1)	6 (14.0)	7 (16.3)	
CT Time from arrest, h	1.2 (0.8–2.6)	1.6 (0.8–3.7)	1.1 (0.8–2.1)	0.074
Neurologic outcome				0.163
Good outcome	27 (31.4)	10 (23.3)	17 (39.5)	

Values are presented as mean ± standard deviation, n (%), median [range], and median (interquartile ranges). SD, standard deviation; GCS, Glasgow coma scale; CT, computer tomography

There was a clear difference in the density of GM and WM between the two CT groups. The GWR-AVE, regardless of the CT scanner used, was 1.256 (IQR, 0.938–1.401) that of the Lightspeed scanner was 1.217 (IQR, 0.938–1.311), and that of the SOMATOM scanner was 1.295 (IQR, 1.169–1.401). Similarly, GWR in different areas all showed similar results (Table 2).

**Table 2:** The density of measured HUs value and GWR according to CT scanner.

	All patients (n = 86)	LightSpeed (n = 43)	SOMATOM (n = 43)	p-value
CN	36.5 (24.3–46.4)	31.8 (24.3–38.7)	41.2 (34.5–46.4)	<0.001
PU	36.4 (24.8–45.3)	32.2 (24.8–38.8)	40.5 (34.5–45.3)	<0.001
PIC	29.0 (22.1–35.3)	26.4 (22.1–32.5)	31.6 (27.2–35.3)	<0.001
CC	28.9 (22.6–34.9)	26.2 (22.6–32.4)	31.5 (26.4–34.9)	<0.001
GWR-CN/PIC	1.255 (0.924–1.432)	1.206 (0.924–1.373)	1.304 (1.104–1.432)	<0.001
GWR-PU/PIC	1.252 (0.943–1.366)	1.221 (0.943–1.366)	1.282 (1.166–1.362)	<0.001
GWR-CN/CC	1.260 (0.933–1.440)	1.213 (0.933–1.318)	1.307 (1.137–1.440)	<0.001
GWR-PU/CC	1.257 (0.952–1.388)	1.223 (0.952–1.326)	1.286 (1.160–1.388)	<0.001
GWR-AVE	1.256 (0.938–1.401)	1.217 (0.938–1.311)	1.295 (1.169–1.401)	<0.001

CN; Caudate nucleus, PU; Putamen, PIC; Posterior internal capsule, CC; Corpus callosum; GWR-AVE, (CN+PU) to (PIC+CC) ratio.

**Fig 2:**
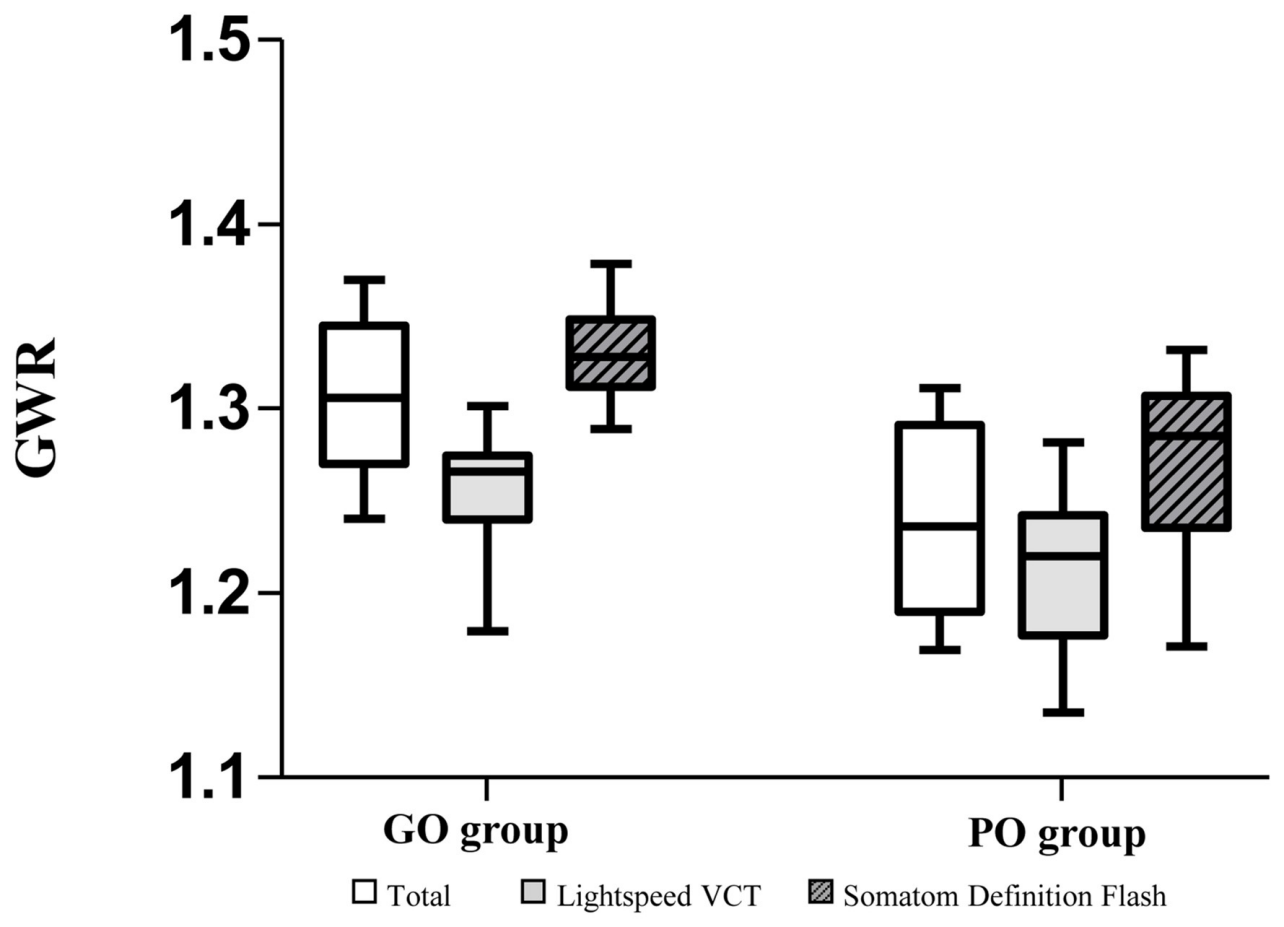
Box-plot is showing the comparison of GWR between each neurologic outcome group. GWR, gray-to-white ratio; GO, good neurologic outcome; PO, poor neurologic outcome.

The analysis of neurological outcomes clearly showed a difference between scanners. In the good outcome analysis, the GWR-AVE of the Lightspeed scanner was 1.266 (IQR, 1.240–1.275), and that of the SOMATOM scanner was 1.328 (IQR, 1.312–1.349). In the poor outcome analysis, the Lightspeed scanner was 1.220 (IQR, 1.177–1.242), and the SOMATOM scanner was 1.285 (IQR, 1.236–1.307) (Table 3 and Fig 2). The cut-off means a value with 100% specificity in the ROC curve analysis that predicts the poor neurological outcome of GWR. The cut-off value of the GWR also showed different results depending on the scanner (Table 4). SOMATOM CT scanner showed a higher cut-off value than the Lightspeed CT scanner. The cut-off value for the entire patient matched the cut-off value for the Lightspeed CT scanner. The Cronbach α of the two researchers was 0.904, with ICCs being excellent.

**Table 3:** Differences in the neurological outcome and GWR for each CT scanner.

	All patients (n = 86)			Good neurologic outcome (n = 27)			Poor neurologic outcome (n = 59)		
	GO	PO	P-value*	Lightspeed (n = 10)	SOMATOM (n = 17)	P-value*	Lightspeed (n = 33)	SOMATOM (n = 26)	P-value*
CN	39.6 (33.2–42.4)	34.5 (30.3–41.0)	0.021	32.4 (31.8–34.3)	41.9 (39.7–43.4)	<0.001	31.0 (29.2–33.7)	41.2 (39.1–42.4)	<0.001
PU	38.8 (33.2–41.3)	34.6 (31.5–40.4)	0.040	32.8 (32.1–34.2)	40.5 (38.7–42.5)	<0.001	32.4 (29.8–33.8)	40.6 (38.6–41.5)	<0.001
PIC	29.0 (26.6–32.1)	27.9 (26.2–32.0)	0.583	26.0 (25.7–27.4)	30.8 (28.9–32.8)	<0.001	26.4 (24.8–27.6)	32.2 (31.6–33.7)	<0.001
CC	29.2 (26.8–31.6)	28.3 (25.8–32.1)	0.387	26.7 (24.8–27.8)	31.1 (28.9–32.9)	<0.001	26.0 (24.3–27.7)	32.2 (30.9–33.3)	<0.001
GWR-CN/PIC	1.321 (1.260–1.353)	1.235 (1.165–1.295)	<0.001	1.269 (1.227–1.290)	1.346 (1.322–1.376)	<0.001	1.182 (1.158–1.250)	1.284 (1.232–1.326)	<0.001
GWR-PU/PIC	1.295 (1.260–1.344)	1.237 (1.193–1.281)	<0.001	1.267 (1.237–1.285)	1.325 (1.294–1.355)	0.001	1.208 (1.182–1.243)	1.270 (1.220–1.295)	0.004
GWR-CN/CC	1.318 (1.270–1.350)	1.240 (1.202–1.279)	<0.001	1.268 (1.210–1.293)	1.346 (1.321–1.377)	<0.001	1.216 (1.170–1.253)	1.293 (1.236–1.329)	<0.001
GWR-PU/CC	1.298 (1.270–1.318)	1.250 (1.207–1.285)	<0.001	1.267 (1.222–1.293)	1.314 (1.295–1.342)	0.001	1.231 (1.184–1.270)	1.276 (1.234–1.300)	0.005
GWR-AVE	1.306 (1.270–1.345)	1.236 (1.190–1.291)	<0.001	1.266 (1.240–1.275)	1.328 (1.312–1.349)	<0.001	1.220 (1.177–1.242)	1.285 (1.236–1.307)	<0.001

GO, good neurologic outcome; PO, poor neurologic outcome; CN, caudate nucleus; PU, putamen; PIC, posterior internal capsule; CC, corpus callosum; GWR, gray to white ratio; GWR-AVE, (CN+PU) to (PIC+CC) ratio.

* Statistical significances were tested by the Mann-Whitney test.

**Table 4:** AUC value and a cut-off value of GWR for each CT scanner.

	All patients (n = 86)			LightSpeed (n = 43)			SOMATOM (n = 43)		
	AUC	cutoff	P-value	AUC	cutoff	P-value	AUC	cutoff	P-value
GWR-CN/PIC	0.791 (0.694–0.888)	1.168	<0.001	0.779 (0.632–0.926)	1.168	0.008	0.826 (0.700–0.951)	1.253	<0.001
GWR-PU/PIC	0.793 (0.695–0.890)	1.197	<0.001	0.782 (0.631–0.933)	1.194	0.008	0.838 (0.714–0.963)	1.224	<0.001
GWR-CN/CC	0.789 (0.684–0.894)	1.149	<0.001	0.768 (0.568–0.968)	1.149	0.011	0.799 (0.666–0.931)	1.265	0.001
GWR-PU/CC	0.761 (0.651–0.870)	1.169	<0.001	0.673 (0.484–0.862)	1.169	0.101	0.799 (0.669–0.929)	1.254	0.001
GWR-AVE	0.812 (0.718–0.907)	1.172	<0.001	0.798 (0.643–0.954)	1.172	0.005	0.855 (0.741–0.969)	1.269	<0.001

The AUC (95% confidence interval) and Cut-off are shown by yielding 100% specificity in each scanner.

AUC, area under the curve; GWR, gray to white ratio; CN, caudate nucleus; PU, putamen; PIC, posterior internal capsule; CC, corpus callosum; GWR-AVE, (CN+PU) to (PIC+CC) ratio.

## Discussion

**Fig 3:**
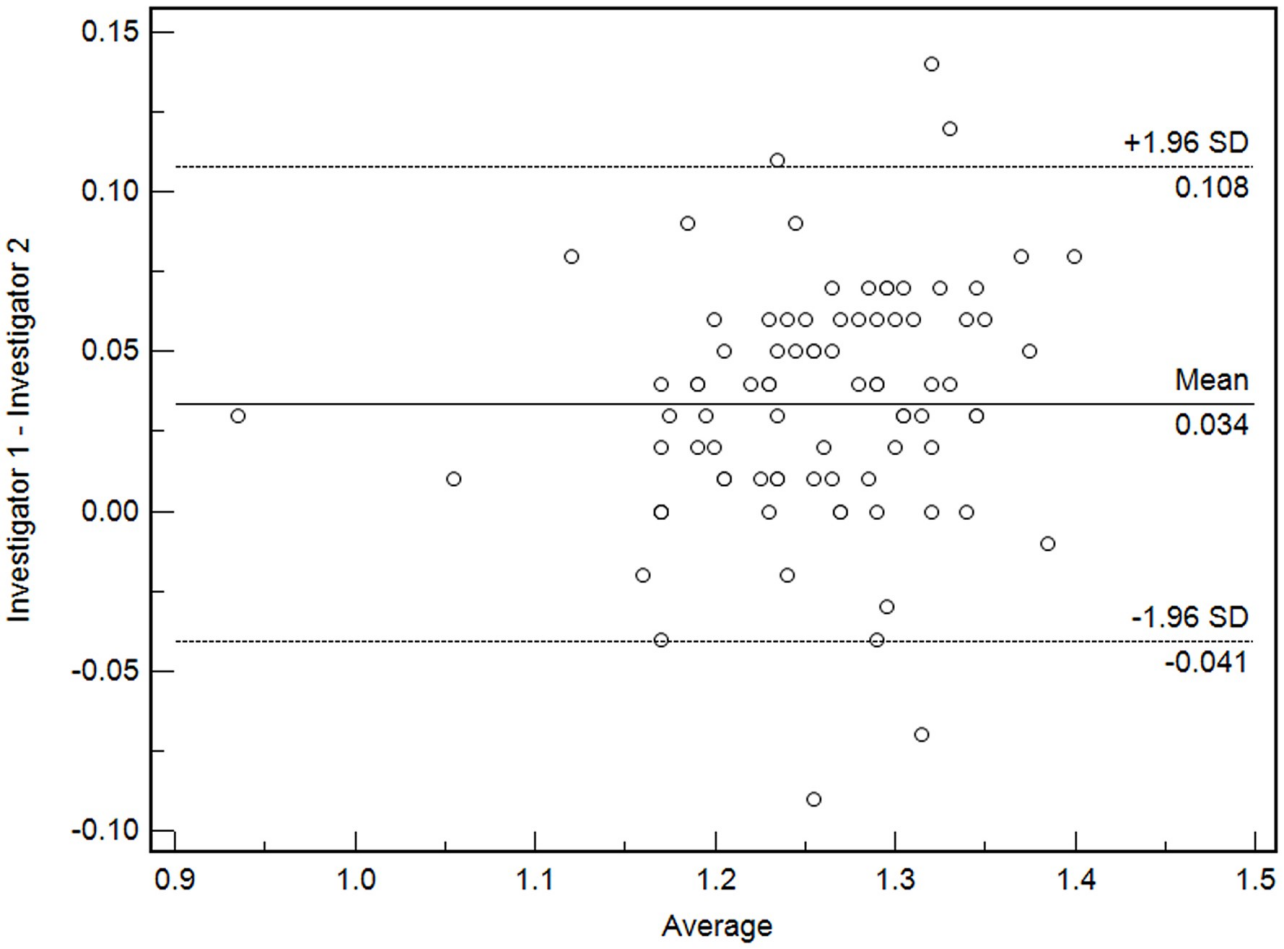
Bland-altman plots of interclass correlation coefficients between investigators.

The authors used color mapping and sectioning methods as in previous studies to minimize errors in the HUs measurement by ROI [19]. The two methods were used because ROI-based analysis uses the average value of the measurement range. Hence, the value changes even if the measurement of the investigator is slightly off [3, 20]. The color mapping method makes the boundary more clear. Additionally, it is not appropriate to measure the ROI in a single area because the HUs value of the measurement zone is not homogeneous. The sectioning method was used to measure three ROI in each zone after each measurement area was divided into three sections, and the average of the nine values obtained was defined as the HUs value in that area [19]. In this study, two researchers measured the ROI by this method, and the ICCs were excellent (Fig 3).

Most previous studies have been retrospective in which only one type of scanner was used. However, there are many kinds of machines used in multi-center studies, and the parameters differ. In a recent study, Gentsch et al. determined a simplified measurement method for the GWR, and the authors commented that three types of CT scanners were used and that the results showed variability [9]. In addition, the radiologist pointed out the variation of scanners and parameters in the commentary for the study analyzing the GWR and neurologic outcome of cardiac arrest patients who performed extracorporeal membrane oxygenation [17]. In this study, different scanners also showed the results of different GWR. In particular, the absolute differences in the GWR-AVE were 0.062 and 0.065 for good and poor neurological outcome, respectively.

These differences can also be misjudged when clinically predicting poor neurologic outcome. When the SOMATOM scanner was applied with a Lightspeed cut-off, the specificity remained 100%, but the sensitivity was reduced. Conversely, applying the cut-off of Somatom to the Lightspeed scanner will predict good neurologic outcome as poor neurologic outcome.

Recently, a multi-center study by Korean Hypothermia Network (KORHN) investigators of PCAS patients receiving TTM showed that the GWR did not correlate with neurologic outcomes [21]. In addition, the poor neurologic outcome prediction sensitivity of the GWR_basal_ ganglia measured with brain CT obtained within one hour of ROSC showed 3.3% at 100% specificity. In contrast, the prediction sensitivity of poor neurologic outcomes in our study, which showed the GWR at the level of the BG at 100% specificity in the CT1 and CT2 groups, was 24.2% and 42.3%, respectively. Furthermore, regardless of the CT scanner used, the sensitivity decreased to 18.6% when all patients were analyzed. In addition, the cut-off value at 100% specificity also differed by 0.097. It matched the value of the Lightspeed scanner, with a low value for the entire patient population regardless of the scanner used. Analyzing data from a multi-center study without considering the scanner used may result in lower sensitivity because GWR converges to the machine’s cut-off value with the lowest cut-off value.

The recent neurological outcome evaluation of patients resuscitated after cardiac arrest involves multimodal approaches, and the GWR is one of them. However, recent GWR studies have tended to report negative results. However, considering the results of this study, the neurologic outcome prediction power of GWR may have been evaluated less than the actual GWR. In addition, recent imaging techniques, such as MRI or CT, undergo a normalization process to compensate for differences in equipment [18, 22–25]. To our knowledge, no study has reported normalization for GWR measurements. This study has several limitations. First, as it was a retrospective study, the same parameters (voltage, thickness, current) of the two CT scans could not be applied. However, all raw data were applied with consistent protocols for each CT. Therefore, the results of this study may reflect the parameter difference. Nevertheless, the results of this study mean that the GWR of the scanner under different conditions may be different. Second, we did not compare the differences between each scanner in the same patient. However, such research design is impossible due to radiation hazards and ethical issues. Third, the purpose of early brain CT scan in cardiac arrest patients is to determine the cause of arrest and determine if it corresponds to an indication of TTM. In addition, recent studies suggest that the GWR of delayed brain CT is suitable for neuroprognostication. In this study, the brain CT scanned after TTM was not analyzed. Fourth, the small sample sizes of single hospitals do not allow the generalization of these results. There is also a potential difference between CT groups in etiology. Therefore, it is necessary to confirm these results in well-designed prospective multi-center studies. Lastly, we did not consider other factors affecting GWR, such as patient age and CT scan time.

## Conclusions

This study showed that inter-scanner variability might be observed in post-cardiac arrest patients who received TTM, and the GWR could vary. Therefore, future research on the GWR may need to incorporate a normalization method to correct for differences between machines.
